# The intracellular domain of homomeric glycine receptors modulates agonist efficacy

**DOI:** 10.1074/jbc.RA119.012358

**Published:** 2021-02-20

**Authors:** Josip Ivica, Remigijus Lape, Vid Jazbec, Jie Yu, Hongtao Zhu, Eric Gouaux, Matthew G. Gold, Lucia G. Sivilotti

**Affiliations:** 1 Department of Neuroscience, Physiology and Pharmacology, Division of Biosciences, University College London, London WC1E 6BT, United Kingdom; 2 Department of Synthetic Biology and Immunology, National Institute of Chemistry, Hajdrihova 19, 1000 Ljubljana, Slovenia; 3 Vollum Institute, Oregon Health & Science University, Portland, Oregon 97239; 4 Howard Hughes Medical Institute, Oregon Health & Science University, Portland, Oregon 97239

**Keywords:** 5-HT_3_, 5-hydroxytryptamine type 3, DMEM, Dulbecco’s modified Eagle’s medium, ECD, extracellular domain, GlyR, glycine receptor, ICD, intracellular domain, *P*_open_, open probability, PDB, Protein Data Bank, pLGIC, pentameric ligand-gated ion channels, TM, transmembrane, zf, zebrafish

## Abstract

Like other pentameric ligand-gated channels, glycine receptors (GlyRs) contain long intracellular domains (ICDs) between transmembrane helices 3 and 4. Structurally characterized GlyRs are generally engineered to have a very short ICD. We show here that for one such construct, zebrafish GlyR_EM_, the agonists glycine, *β*-alanine, taurine, and GABA have high efficacy and produce maximum single-channel open probabilities greater than 0.9. In contrast, for full-length human *α*1 GlyR, taurine and GABA were clearly partial agonists, with maximum open probabilities of 0.46 and 0.09, respectively. We found that the elevated open probabilities in GlyR_EM_ are not due to the limited sequence differences between the human and zebrafish orthologs, but rather to replacement of the native ICD with a short tripeptide ICD. Consistent with this interpretation, shortening the ICD in the human GlyR increased the maximum open probability produced by taurine and GABA to 0.90 and 0.70, respectively, but further engineering it to resemble GlyR_EM_ (by introducing the zebrafish transmembrane helix 4 and C terminus) had no effect. Furthermore, reinstating the native ICD to GlyR_EM_ converted taurine and GABA to partial agonists, with maximum open probabilities of 0.66 and 0.40, respectively. Structural comparison of transmembrane helices 3 and 4 in short- and long-ICD GlyR subunits revealed that ICD shortening does not distort the orientation of these helices within each subunit. This suggests that the effects of shortening the ICD stem from removing a modulatory effect of the native ICD on GlyR gating, revealing a new role for the ICD in pentameric ligand-gated channels.

Glycine receptors (GlyRs) belong to the superfamily of pentameric ligand-gated ion channels (pLGIC). Like another member of this group, GABA receptors, GlyRs are permeable to anions and mediate inhibitory synaptic currents, but these are faster than those mediated by GABA and are particularly important in the spinal cord and brainstem. In heterologous expression systems, functional GlyRs can be assembled as homomers of *α* subunits or as heteromers of *α* and *β* subunits and these heteromeric receptors are thought to be the synaptic form of GlyRs in the adult mammalian central nervous system (1).

GlyR subunits follow the general fold of the pLGIC family and each subunit has an extracellular domain (ECD), a transmembrane domain (TMD) formed by four *α*-helices (TM1–TM4) and a large intracellular domain (ICD) between the TM3 and TM4 helices. The neurotransmitter/agonist-binding sites are at the interfaces between the ECDs of adjacent subunits, and the pore, with its activation and desensitization gates, is formed by the TM2 helices. These features were confirmed specifically for GlyR by the solution of crystal structures of homomeric human *α*3 GlyR and cryo-EM structures of zebrafish *α*1 GlyR (2, 3, 4). Neither of these sets of data gives us information about the ICD, because both were obtained from constructs where the ICD (which is up to ∼80 amino acids long in GlyR) had been replaced by an AGT tripeptide linker.

Ablation of the ICD is an almost universal feature in structural work on pLGICs, and the first high resolution structures of channels in this superfamily were obtained from prokaryotic pLGICs, such as ELIC and GLIC (5, 6), where the TM3–TM4 linker is naturally very short.

The main functional features of pLGICs are thought to survive the replacement of the native ICD with the heptapeptide TM3–TM4 linker found in GLIC or with an AGT tripeptide, and this has been shown in 5-HT_3_ receptors, GABA *ρ* channels (7), GluCl (8), and GlyRs (2, 3, 9).

As structural work is undertaken to explore the determinants of agonist efficacy in pLGICs, we tried to identify GlyR agonists that are reliably partial on GlyR constructs that could be characterized in structural investigations, and started with GlyR_EM_, the zebrafish *α*1 construct used by Du *et al.* (2).

Our single-channel measurements of maximum open channel probability for a set of four agonists, from the full agonist Gly to the weak partial agonist GABA, show that the efficacy of partial agonists is much higher in the ICD-less GlyR_EM_ construct than in the human WT *α*1 homomeric GlyR. Although the sequences of the human and zebrafish GlyR subunits have a few differences, we show that most of the increase in agonist efficacy was due to the drastic shortening of the ICD. This increase could be reproduced in the human GlyR by ICD excision and reversed in the zebrafish receptor by ICD re-insertion, pointing out to a role for the ICD in modulating the efficacy of agonist gating.

## Results

### Partial agonists are more efficacious in zebrafish recombinant α1 GlyR_EM_, a construct with a shortened ICD, than in human α1 GlyRs

**Figure 1:**
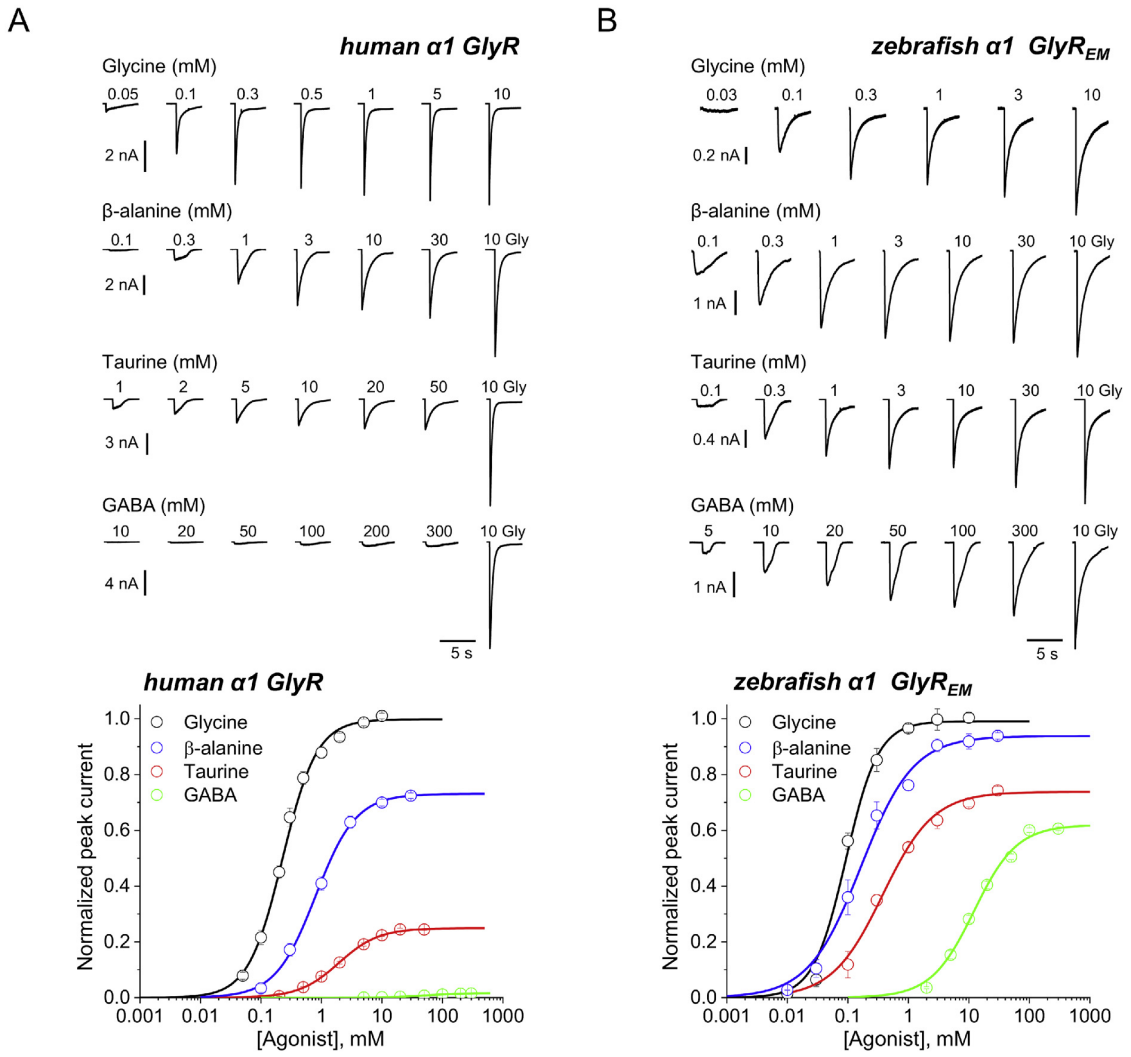
**GlyR agonist efficacy is lower in human***α***1 GlyR than in zebrafish***α***1 GlyR**_**EM**_**, a channel from which the ICD has been excised.***A* and *B, upper panels*, whole cell current responses to the application of glycine, *β*-alanine, taurine, and GABA by U-tube to HEK 293 cells expressing human *α*1 GlyR (*A*) or zebrafish *α*1 GlyR_EM_ (*B*). *A* and *B, lower panels*, averaged concentration-response curves of glycine (*black*), *β*-alanine (*blue*), taurine (*red*), and GABA (*green*) in human *α*1 GlyR (*A*) and zebrafish *α*1 GlyR_EM_ (*B*). Each dose-response curve is constructed by pooling individual concentration-response curves obtained in different cells (*n* = 4 –14, see Table 1). *Error bars* represent S.E. Responses were normalized to the response to 10 mm glycine in each cell.

Fig. 1 shows whole cell responses to agonists of recombinant homomeric *α*1 GlyR expressed in HEK293 cells. The data in *panel A* are from human WT GlyR, and those in *panel B* are from zebrafish GlyR_EM_, a receptor construct for cryoEM structure determination that is engineered to have a tripeptide linker in place of the ICD. We tested as agonists glycine, β-alanine, taurine, and GABA, and applied them by U-tube at concentrations up to 300 mm. The time course of the current responses (*top panels*) was similar for the two receptors and the different agonists and showed the features expected of agonist responses from a pLGIC. As agonist concentrations increased, responses increased in amplitude, reaching their peak more quickly and also declining from this peak more quickly because of faster desensitization.

Peak current responses were normalized to the response to a 10 mm glycine standard in each cell (last response in each set of traces) and plotted as dose-response curves in the bottom two panels (see Table 1 for the parameters of Hill equation fits to the data). Zebrafish GlyR_EM_ was more sensitive to agonists, and EC_50_ values of each agonist were between 2.5- and 5-fold lower for this receptor than for the human GlyR *α*1 (Table 1). The rank order of potency and efficacy was similar for the two receptors, with glycine >*β*-alanine > taurine > GABA (and is in line with the literature for human GlyR and zebrafish GlyR, (10, 11, 12, 13)). However, the two receptors were strikingly different when the size of the agonist maximum responses was compared. Thus, in human WT *α*1 GlyR, *β*-alanine is clearly a partial agonist that elicits a maximum response that is 73% of that of glycine, taurine is even weaker (25%), and GABA is almost completely ineffective as agonist (1.7%). In contrast to that, on the zebrafish *α*1 GlyR_EM_, *β*-alanine is almost a full agonist, eliciting a maximum response that is 93% of that to glycine, and both taurine and GABA are much more efficacious (74 and 62%) than on human receptors (*p* < 0.005 for all agonists; see Table 1 and Table S1).

**Table 1:** Whole cell recordings

Whole cell parameters					
GlyR	Glycine	*β*-Alanine	Taurine	GABA	*I*_max_ glycine (nA)
**Human *α*1 GlyR**					
*I*_rel_	–	0.73 ± 0.11	0.25 ± 0.09	0.017 ± 0.020	13.2 ± 3
EC_50_ (*μ*M)	240 ± 60	860 ± 250	2,000 ± 400	61,000 ± 14,000	
*n*_H_	1.57 ± 0.22	1.5 ± 0.24	1.30 ± 0.11	1.32 ± 0.07	
*n*	10	6	7	5	
**Zebrafish *α*1 GlyR**					
*I*_rel_	–	0.84 ± 0.09	0.40 ± 0.20	0.14 ± 0.13	9.0 ± 1.4
EC_50_ (*μ*M)	190 ± 60	340 ± 190	1050 ± 220	28,400 ± 3,000	
*n*_H_	1.9 ± 0.3	1.34 ± 0.19	1.26 ± 0.12	1.59 ± 0.17	
*n*	8	10	6	8	
**Human *α*1 GlyR ΔICD**					
*I*_rel_	–	0.85 ± 0.04	0.55 ± 0.07	0.07 ± 0.03	2.8 ± 1.0
EC_50_ (*μ*m)	140 ± 20	450 ± 190	1500 ± 210	33,200 ± 11,000	
*n*_H_	1.43 ± 0.38	1.25 ± 0.24	1.05 ± 0.16	1.42 ± 0.17	
*n*	5	4	5	6	
**Human *α*1 GlyR ΔICD + TM4 zebrafish**					
*I*_rel_	–	0.86 ± 0.07	0.56 ± 0.06	0.10 ± 0.04	2.2 ± 0.6
EC_50_ (*μ*m)	180 ± 32	320 ± 70	1480 ± 570	93,200 ± 45,000	
*n*_H_	1.19 ± 0.20	1.56 ± 0.24	1.03 ± 0.19	0.98 ± 0.11	
*n*	8	7	7	5	
**Zebrafish *α*1 GlyR_EM_**					
*I*_rel_	–	0.93 ± 0.03	0.74 ± 0.13	0.62 ± 0.12	3.3 ± 0.8
EC_50_ (*μ*m)	92 ± 10	170 ± 90	390 ± 140	12,400 ± 4000	
*n*_H_	1.6 ± 0.4	1.17 ± 0.15	1.03 ± 0.11	1.26 ± 0.22	
*n*	4	6	5	14	

Agonist efficacy and sensitivity values were estimated from the fit of dose-response curves and are presented as mean ± S.D. *I*_rel_, maximum current normalized to that of glycine in the same cell.

### Partial agonists produce a higher single-channel maximum open probability in zebrafish α1 GlyR_EM_ than in human α1 GlyRs

**Figure 2:**
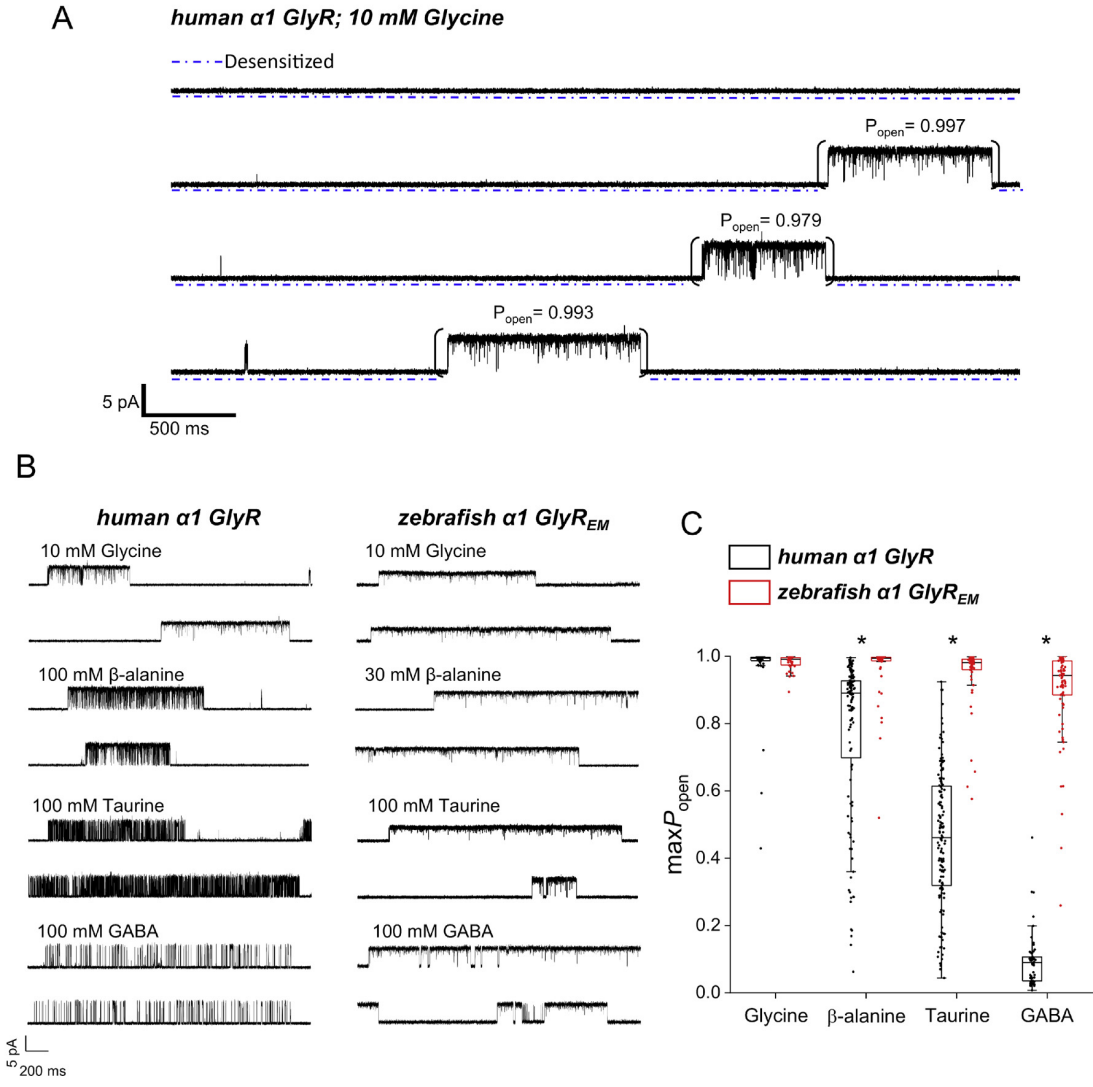
**Single-channel recordings show that agonists are more efficacious on zebrafish***α***1 GlyR**_**EM**_**compared with human GlyR***α***1.***A*, example of a cell-attached single-channel recording from human *α*1 GlyR activated by 10 mm glycine in the recording electrode. Three clusters of single-channel activity are separated by long desensitized intervals (*dashed lines under* the trace). *P*_open_ values shown *above* each cluster were obtained as ratios between cluster open time and cluster duration. Long desensitized intervals were not included in the analysis. *B,* single-channel activity evoked by saturating concentrations of glycine, *β*-alanine, taurine, and GABA for human *α*1 GlyR (*left panel*) and zebrafish *α*1 GlyR_EM_ (*right panel*). *C*, boxplot showing maximum *P*_open_ of glycine, *β*-alanine, taurine, and GABA for human *α*1 GlyR (*black*, *left hand side* in each pair) and zebrafish *α*1 GlyR_EM_ (*red*, *right hand side*). Each point is a *P*_open_ value obtained from a cluster of single-channel activity in the presence of 10 mm glycine, 100 mm*β*-alanine, 100 mm taurine, or 100 mm GABA. *Boxes* show the 25th and 75th percentiles, and *whiskers* the furthest points that fall within 1.5 times of the interquartile range from the 25th to 75th percentiles. The *horizontal line* in each box shows the median. *Asterisks* denote significant differences in randomization tests, two-tail, unpaired; 10,000 iterations *p* < 0.005.

To obtain better estimates of agonist efficacy in the two GlyRs, we switched to single-channel experiments, recording the effect of saturating concentrations of each agonist. Fig. 2*A* shows a typical cell-attached single-channel trace at high glycine concentration. Most of the time the patch is silent because all the channels in the patch are desensitized (*dashed line* under the trace). Every now and again a channel emerges from desensitization, gives rise to a “cluster,” a group of openings (the upwards deflections in the trace) separated by short shut times, and desensitizes again. During a cluster, the channel open probability is high enough that we can be sure that only one channel is active (no double openings are seen) and we can measure the maximum open probability produced by the agonist on a single channel molecule (*cf*. values marked on top of each cluster). This is a true equilibrium measurement in the absence of desensitization, it is an absolute measurement and can be obtained for all agonists, including glycine. This is a considerable advantage, *cf*. whole cell experiments, where peak currents are the expression of concurrent activation and desensitization (to an extent that depends on actual rate of agonist exchange at the receptor) and the responses have to be normalized to a glycine standard. Another advantage of single-channel recording is that very small amounts of agonist are needed for each cell-attached experiment. This made it possible to use our limited supply of purified agonists, which contain less than 1 part contaminant glycine in 600,000.

Fig. 2*B* shows representative single channel cluster openings produced by saturating concentrations of agonists on the human *α*1 GlyR (on the *left*) and on the zebrafish GlyR_EM_ (on the *right*). In both GlyRs, the clusters produced by glycine (*top two traces* and Fig. 2*A*) have a high open probability, with relatively few short shuttings, as expected for a full agonist. Effectively, receptors bound to glycine are either open or desensitized. The situation is different for the other agonists. Traces from the human *α*1 GlyR show a clear gradient in open probability and agonist efficacy: there is a visible increase in the proportion of shut time in the cluster, as we move from glycine to *β*-alanine to taurine and efficacy decreases. The clusters look darker because of the number of shuttings and brief openings, which are unresolved to the eye at this scale. In the case of GABA (*bottom traces*), the *P*_open_ is even lower, the shut times are longer and clearly visible between the short openings in the cluster. Fig. 2*C* is a plot of all cluster open probability values, where each value is represented by a point (the same data are summarized in Table 2). This plot shows that in human *α*1 GlyRs the maximum *P*_open_ declines progressively from glycine to *β*-alanine, taurine, and GABA, with averages of 0.97, 0.78, 0.46, and 0.09. This pattern is clear despite substantial cluster-to-cluster *P*_open_ variability, especially for the agonists with intermediate efficacy, such as *β*-alanine and taurine. The same experiments in zebrafish GlyR_EM_ (Fig. 2*B*, *right*) produced a very different pattern, where all agonists produced similar high open probability clusters, which looked similar to glycine clusters. Plotting the *P*_open_ values in the graph in Fig. 2*C* (*red points*) shows that the average maximum *P*_open_ values were around or above 0.90, with agonists glycine, *β*-alanine, taurine, and GABA producing 0.98, 0.97, 0.95, and 0.90 *P*_open_, respectively. There was no difference in the maximum *P*_open_ of glycine on the two receptors (*p* = 0.34). However, all agonists that were partial on human *α*1 GlyRs produced a significantly higher maximum *P*_open_ for zebrafish GlyR_EM_ (*p* < 0.005). As the average *P*_open_ increased, the extent of cluster-to-cluster variability decreased, also resembling the properties of the full agonist glycine. Thus the single-channel experiments strongly confirmed that agonists that are partial on human *α*1 GlyR are much more efficacious on zebrafish GlyR_EM_ channels.

**Table 2:** Single channel parameters measured for five GlyRs

Single-channel parameters					
GlyR	Glycine	*β*-Alanine	Taurine	GABA	Amplitude (pA)
**Human *α*1 GlyR**					
max*P*_open_	0.97 ± 0.11	0.78 ± 0.24	0.46 ± 0.20	0.09 ± 0.08	5.96 ± 0.70
*n*_patches_ (*n*_clusters_)	5 (47)	5 (124)	11 (144)	13 (59)	
**Zebrafish***α***1 GlyR**					
max*P*_open_	0.97 ± 0.05	0.91 ± 0.21	0.66 ± 0.24	0.40 ± 0.25	5.45 ± 0.92
*n*_patches_ (*n*_clusters_)	10 (48)	7 (30)	10 (71)	11 (83)	
**Human a1 GlyR ΔICD**					
max*P*_open_	0.99 ± 0.01	0.92 ± 0.07	0.90 ± 0.10	0.70 ± 0.21	4.11 ± 0.72
*n*_patches_ (*n*_clusters_)	5 (26)	6 (26)	10 (33)	4 (37)	
**Human α1 GlyR ΔICD + TM4 zebrafish**					
max*P*_open_	0.98 ± 0.02	0.88 ± 0.16	0.89 ± 0.12	0.73 ± 0.24	3.63 ± 0.88
*n*_patches_ (*n*_clusters_)	4 (16)	7 (29)	6 (41)	4 (43)	
**Zebrafish *α*1 GlyR ΔICD**					
max*P*_open_	0.99 ± 0.02	0.98 ± 0.05	0.93 ± 0.13	0.89 ± 0.13	4.74 ± 1.10
*n*_patches_ (*n*_clusters_)	5 (32)	9 (25)	7 (26)	5 (23)	
**Zebrafish***α***1 GlyR**_**EM**_					
max*P*_open_	0.98 ± 0.02	0.97 ± 0.08	0.95 ± 0.10	0.90 ± 0.14	5.54 ± 1.59
*n*_patches_ (*n*_clusters_)	10 (42)	9 (54)	17 (55)	7 (77)	

The values are presented as mean ± S.D.

### Excising the ICD from the human α1 GlyR increases agonist efficacy

**Figure 3:**
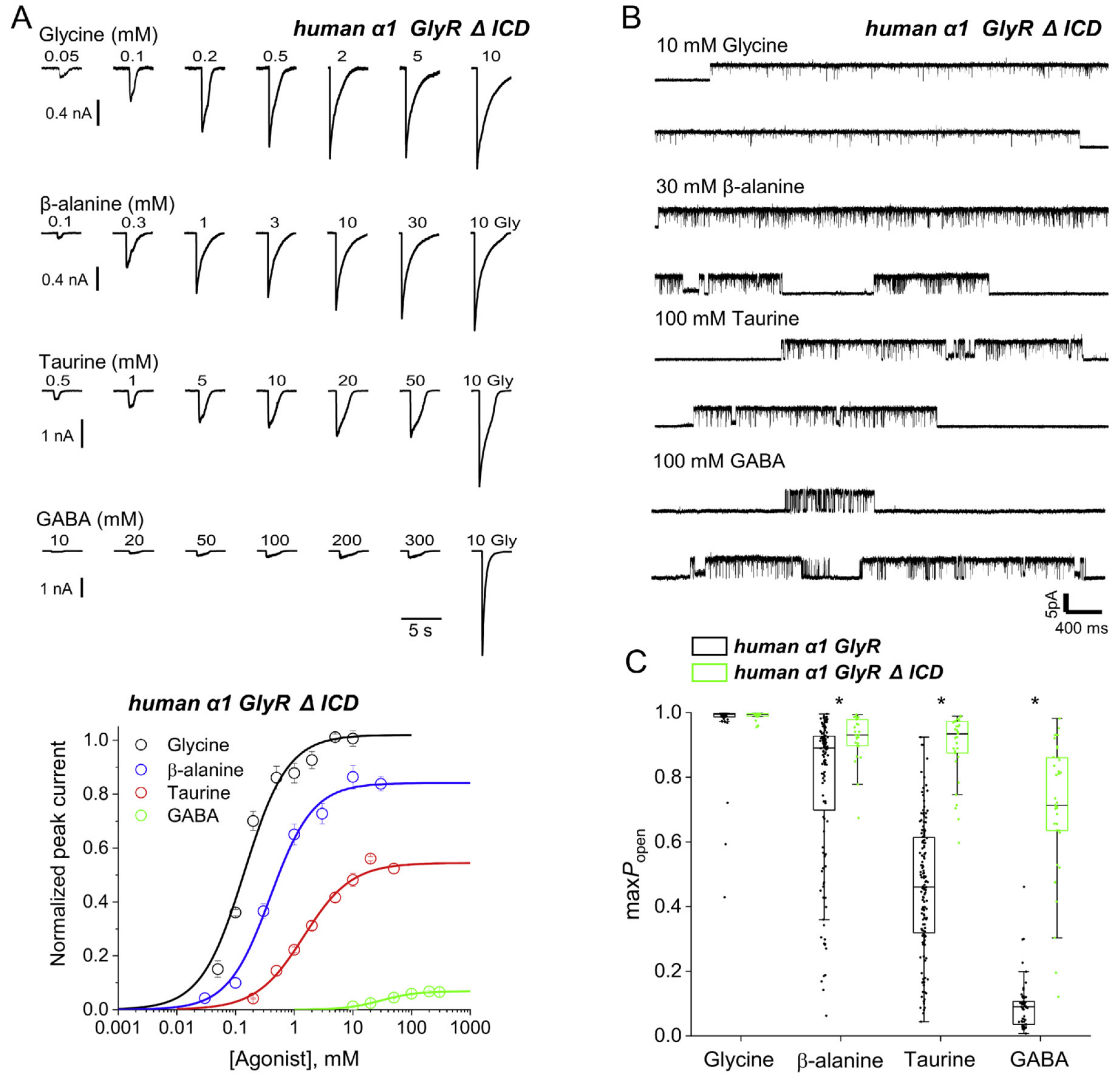
**Removing the ICD loop from the human *α*1 GlyR increases agonist efficacy.***A*, *upper panel*, whole cell current responses to U-tube application of glycine, *β*-alanine, taurine and GABA to HEK 293 cells expressing human *α*1 GlyR Δ ICD. *A, lower panel*. Averaged concentration-response curves to glycine (*black*), *β*-alanine (*blue*), taurine (*red*) and GABA (*green*) on human *α*1 GlyR Δ ICD. Each curve is constructed from pooling individual concentration-response curves obtained in different cells (*n* = 4-6, see Table 1). *Error bars* represent S.E. Responses are normalised to the response to 10 mm glycine in each cell. *B,* cell-attached recordings of clusters of single-channel activity evoked in human *α*1 Δ ICD by saturating agonist concentrations (10 mm glycine, 30 mm*β*-alanine, 100 mm taurine, 100 mm GABA). *C*, boxplot of maximum *P*_open_ values produced by at saturating agonist concentrations for human *α*1 GlyR (*black, left hand side* in each pair) and human *α*1 GlyR ΔICD (*green, right hand side* in each pair). Each point is the *P*_open_ value from a cluster of single-channel activity. *Boxes* and *whiskers* show the 25th and 75th percentiles and the furthest points that fall within 1.5 times of the interquartile range from the 25th to 75th percentiles, respectively. The *horizontal line* in the box is the median. *Asterisks* denote significant differences in randomization tests (two tail, unpaired; 10000 iterations; *p* < 0.005).

These differences seemed surprising, given that the two receptors have a high degree of sequence similarity. Apart from the ICD, the human *α*1 GlyR and zebrafish *α*1 GlyR_EM_ differ only in 25 amino acids (12 are in ECD, one in TM1, 2 in TM3, and 10 in TM4, and none of the differences are in the residues involved in binding glycine (14) (see Fig. S1)). The most obvious difference between the two GlyRs is the ICD, more specifically its absence in GlyR_EM_. Thus we generated a new GlyR construct by replacing the large ICD from the human GlyR *α*1 with a short tripeptide linker (AGT), the same that was used as TM3–TM4 linker in GlyR_EM_ (see Fig. S1). Fig. 3 shows our characterization of this human *α*1 GlyR ΔICD receptor.

ICD excision had no apparent effect on the time course of agonist whole-cell current responses (Fig. 3*A*). However, there was an obvious change in the concentration-response curves, where the maximum responses to taurine and GABA were increased, to 55 and 7% of the glycine maximum (*cf*. 25 and 1.7% in WT, *p* < 0.005, respectively; Table 1). Maximum glycine responses for the human *α*1 GlyR ΔICD were significantly smaller (*p*<0.005) than for WT, suggesting that constructs with a short ICD either have a low expression (15) or have a toxic effect for cells that express them at high levels. Experiments with single- channel recordings proved to be challenging with the human *α*1 GlyR ΔICD constructs, because in the majority of patches no channel activity was detected. We hypothesized that the presence of glycine (400 *μ*M) in our standard (Dulbecco’s modified Eagle’s medium (DMEM)) culture medium reduced the survival of high expressing cells. To increase the success rate of the single channel recordings, we switched the growth media to MEM (Gibco, 11095080), which does not contain glycine in its formulation.

The increase in agonist efficacy produced by ICD excision was confirmed by single-channel recordings: the traces in Fig. 3*B* show that in the human *α*1 GlyR ΔICD all agonists produced long, high open probability clusters, where openings were separated by short unresolved shuttings. These clusters were similar to those observed with glycine in WT receptors and to those observed with all agonists in zebrafish GlyR_EM_. The boxplots in Fig. 3*C* provide an overall view, and compare cluster *P*_open_ values for the WT GlyR (*black*) and GlyR ΔICD (*green*). The increase in open probability after ICD excision are very clear for all agonists except glycine (*p* = 0.34), which was already a very efficacious agonist on WT GlyR, with a *P*_open_ of 0.97 and 0.99 in WT GlyR and GlyR ΔICD, respectively. The increase in maximum *P*_open_ was large for the two agonists with lower efficacy in WT, taurine, and GABA whose maximum *P*_open_ increased from 0.46 to 0.90 (*p* < 0.005) and from 0.09 to 0.70 (*p* < 0.005), respectively (Fig. 2*C*, Table 2). These higher values approach, but do not quite reach, those observed in zebrafish *α*1 GlyR_EM_, where GABA produced a maximum *P*_open_ of 0.90.

### Reinstating the native intracellular domain lowers agonist efficacy in zebrafish GlyR

**Figure 4:**
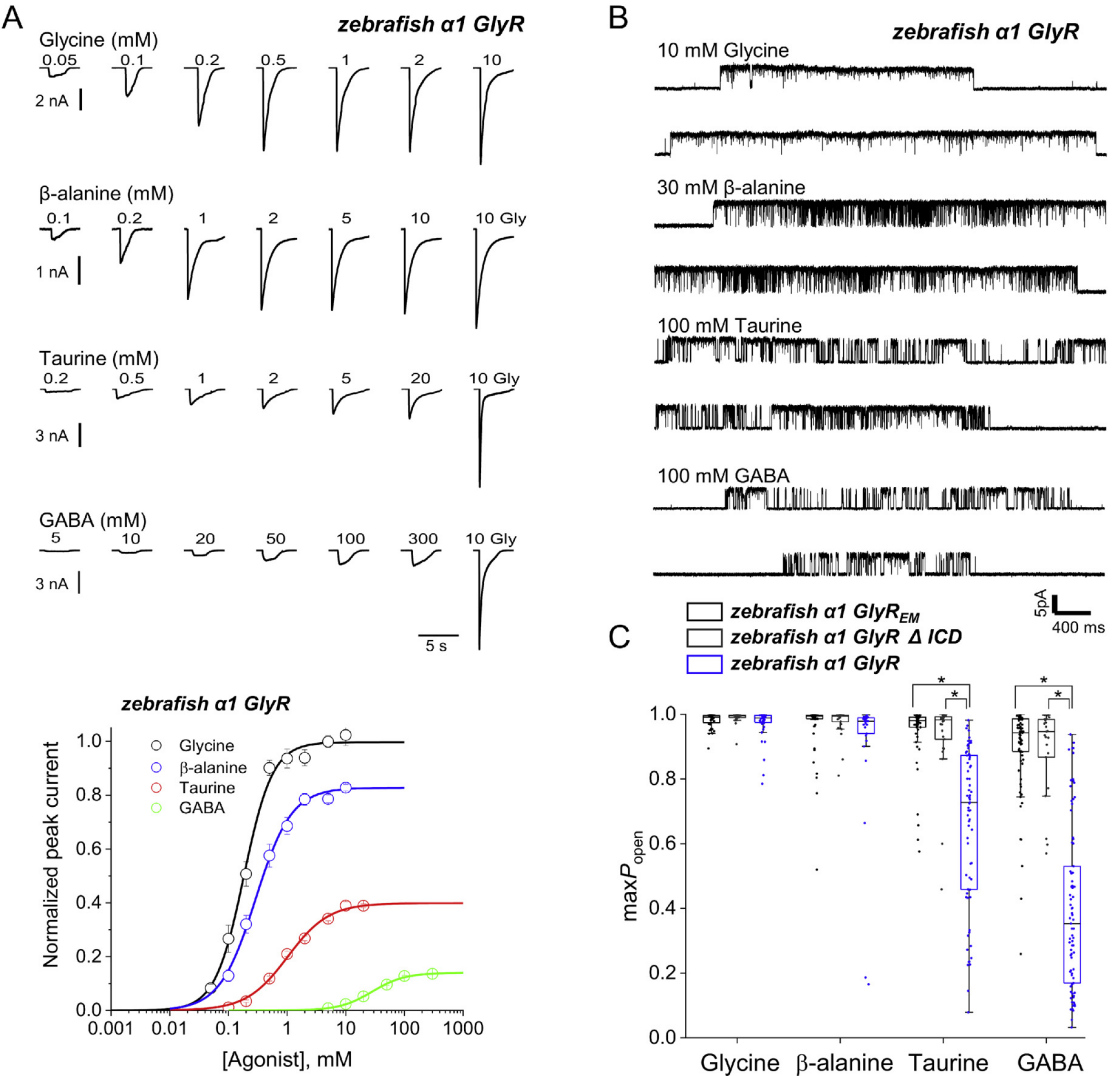
**Reinstating the WT ICD in zebrafish GlyR_EM_ decreases agonist efficacy.***A*, *upper panel*, whole cell current responses to glycine, *β*-alanine, taurine, and GABA of zebrafish WT *α*1 GlyR. *A*, *lower panel*, averaged concentration-response curves to glycine (*black*), *β*-alanine (*blue*), taurine (*red*), and GABA (*green*) on zebrafish *α*1 GlyR. Each curve is constructed from pooling individual concentration-response curves obtained in different cells (*n* = 6–10). *Error bars* represent S.E. Responses are normalized to the response to 10 mm glycine in each cell. *B*, cell-attached recordings of clusters of zebrafish *α*1 single-channel activity evoked by saturating agonist concentrations (10 mm glycine, 30 mm*β*-alanine, 100 mm taurine, 100 mm GABA). *C*, boxplot of the maximum *P*_open_ values produced by saturating concentrations of different agonists for zebrafish *α*1 GlyR_EM_ (*black*, *left hand* side), zebrafish *α*1 GlyR ΔICD (*dark gray* in the *middle*), and zebrafish *α*1 GlyR (*blue*, *right hand side*). Each point is a *P*_open_ value from a cluster of single-channel activity. *Boxes* and *whiskers* show the 25th and 75th percentiles and the furthest points that fall within 1.5 times of the interquartile range from the 25th to 75th percentiles, respectively. The *horizontal line* in the box is the median. *Asterisks* and *brackets* denote differences that reached statistical significance (randomization test, two tail, unpaired; 10,000 iterations; *p* < 0.005).

If excising the ICD was responsible for much of increased agonist efficacy observed in GlyR_EM_, we would expect partial agonists to be less efficacious in WT zebrafish receptors that carry a normal amino acid ICD (almost 70 residues long). Fig. 4 shows that this is exactly what we found. In the zebrafish WT *α*1 GlyR, the time course of agonist-evoked current responses was the same as in the other receptors we examined (*top panel* of Fig. 4*A*). However, the dose-response curves (*bottom panel* of Fig. 4*A*) show that in zebrafish WT *α*1 GlyR the maximum responses to partial agonists (relative to glycine) were smaller, particularly for taurine and GABA (compare with Fig. 1*B*). This decrease in efficacy was associated with a consistent decrease in agonist sensitivity, producing EC_50_ values ∼2-fold higher (see results of the Hill equation fits, Table 1, Fig. S1). As we observed for human GlyRs with and without ICD, the absolute amplitude of maximum glycine currents (*I*_max_) was higher in the zebrafish GlyR with its native ICD (Table 1), again suggesting that the native ICD supports receptor trafficking and expression levels.

Results from single-channel recordings (Fig. 4*B*) confirmed the whole cell data. The clusters of openings at high agonist concentrations appear different for the different agonists and reach mean maximum *P*_open_ values of 0.97, 0.91, 0.66, and 0.40, for glycine, *β*-alanine, taurine, and GABA, respectively. The boxplots in Fig. 4*C* show markedly lower (*p* < 0.005) efficacy for taurine and GABA in the WT GlyR (*blue*) *versus* GlyR_EM_ (*black*). Thus, the presence or absence of a long intracellular domain affects agonist efficacy in the GlyRs from both species.

As the sequences in the alignment in Fig. S1 show, GlyR_EM_ differs from the WT zebrafish GlyR not only in the absence of the ICD, but also in the presence of a thrombin cleavage site at the C terminus. As a further control, we have measured agonist maximum *P*_open_ in a zebrafish receptor in which we have replaced the ICD with the AGT tripeptide. The values we recorded in this receptor (*dark gray* in Fig. 4*C*) were indistinguishable from those measured in GlyR_EM_ (*p* < 0.005).

Although the pattern of agonist efficacy in the full-length zebrafish GlyR resembles that in the human *α*1 GlyR, it is not identical, and partial agonists remain somewhat more efficacious in zebrafish GlyRs (Table 2). The biggest difference is seen for GABA, which in zebrafish *α*1 GlyR produced a maximum *P*_open_ ∼4 times higher than in human *α*1 GlyR (*p* < 0.005; Figs. 2*C* and 4*C*; similar results were obtained at whole-cell level, Figs. 1*A* and 4*A*, and for receptors in which the ICD was excised, see Figs. 2*C* and 3*C*). This is not surprising, as we would expect agonist efficacy to be affected also by receptor differences in domains other than the ICD.

### Exchanging the TM4 domain between zebrafish and human α1 GlyRs

As shown in Fig. S1, many of the differences between zebrafish and human GlyR outside the ICD are in the TM4 helix (10 residues) and in the short C terminus at its end (4 residues).

The TM4 helix is relatively poorly conserved across different isoforms of GlyR within the same species, and differences in TM4 have been proposed to be responsible for the much lower efficacy of partial agonists *β*-alanine and taurine on *α*3 *versus α*1 GlyR (16).

**Figure 5:**
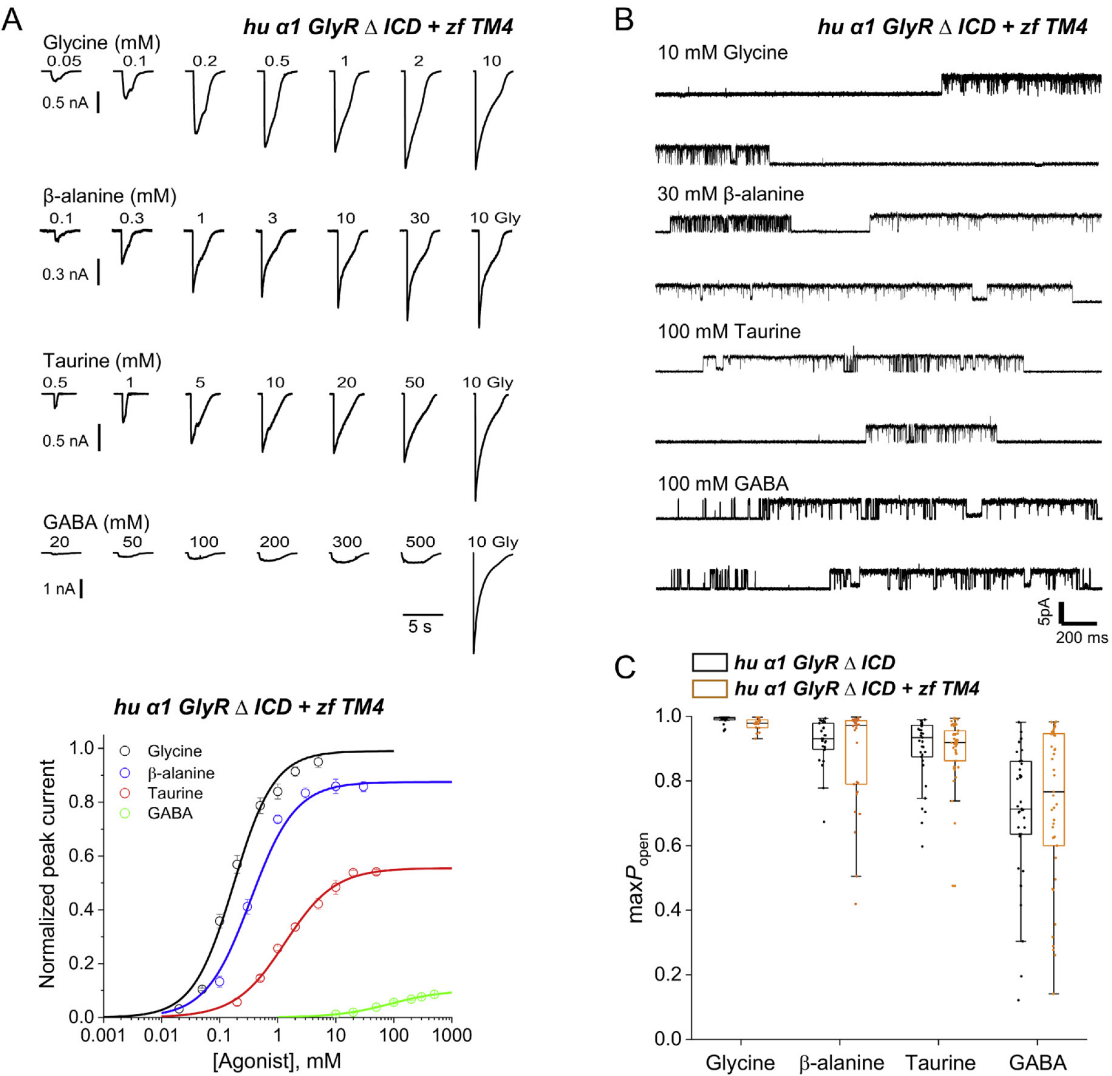
**Replacing the TM4 domain of human GlyR***α***1** Δ**ICD with that of zebrafish GlyR does not increase agonist efficacy.***A*, *upper panel*, whole-cell current responses to U-tube application of glycine, *β*-alanine, taurine, and GABA to HEK 293 cells expressing human *α*1 GlyR ΔICD + zf TM4. *A*, *lower panel*, averaged concentration-response curves to glycine (*black*), *β*-alanine (*blue*), taurine (*red*), and GABA (*green*) on human *α*1 GlyR ΔICD+zf TM4. Each curve is constructed from pooling 5 to 8 curves obtained in different cells. *Error bars* represent S.E. Responses are normalized to the response to 10 mm glycine in each cell. *B,* cell-attached recordings of clusters of openings of human *α*1 GlyR ΔICD + zf TM4 evoked by saturating agonist concentrations (10 mm glycine, 30 mm*β*-alanine, 100 mm taurine, 100 mm GABA). *C,* boxplot showing maximum *P*_open_ values obtained at saturating concentrations of four different agonists (as in *panel B*) for human *α*1 GlyR ΔICD (*black*, *left hand side* in each pair) and human *α*1 GlyR ΔICD + zf TM4 (*orange*, *right hand side* in each pair). Each point is the *P*_open_ value from a cluster of single-channel activity. *Boxes* and *whiskers* show the 25th and 75th percentiles and the furthest points that fall within 1.5 times of the interquartile range from the 25th to 75th percentiles, respectively. The *horizontal line* in the box is the median. None of the differences in open probability between constructs reached significance.

Rather than selecting specific point mutations, we spliced the whole of the TM4 together with the C terminus from WT zebrafish GlyR into the human *α*1 GlyR ΔICD to produce a chimeric GlyR (human *α*1 GlyR ΔICD + zebrafish TM4). Whole-cell dose-response curves for our standard group of agonists glycine, *β*-alanine, taurine, and GABA, are shown in Fig. 5*A*. Maximum currents produced by 10 mm glycine for the chimera were the smallest of all the constructs, suggesting particularly low levels of expression (Table 1), and it was difficult to obtain well-determined dose-response curves for the weakest partial agonist, GABA. With this limitation, insertion of the zebrafish TM4 and C terminus did not change appreciably the apparent efficacy of partial agonists, as the maximum currents for the partial agonists relative to glycine were very similar in the chimeric GlyR and in human *α*1 GlyR ΔICD, with 0.86, 0.56, and 0.1 for *β*-alanine, taurine, and GABA, respectively (*cf*. 0.85, 0.55, and 0.07 in human *α*1 GlyR ΔICD, Table 2; see Figs. 5*A* and 3*A*). The cell-attached single-channel measurements in the chimera (*orange*) and human *α*1 GlyR ΔICD (*black*; Fig. 5*C*) confirm this finding is robust as they gave maximum *P*_open_ values that were indistinguishable, at 0.98, 0.88, 0.89, and 0.73, for the chimera and 0.99, 0.92, 0.90, and 0.70 for the *α*1 GlyR ΔICD for glycine, *β*-alanine, taurine, and GABA, respectively (differences did not reach statistical significance). Thus, on the background of an ICD-less receptor, splicing the zebrafish TM4 and C terminus into the human GlyR had no clear effect on efficacy.

In the zebrafish GlyR ΔICD we had found the maximum *P*_open_ to GABA to be similar to that of the other agonists at about 0.90, but this was not the case for the human *α*1 GlyR ΔICD + zebrafish TM4, where it remains well below the value for taurine. The two receptors differ from one another in the extracellular domain, 1 amino acid in TM1 and in 2 amino acids at the cytoplasmic end of TM3.

## Discussion

Our study shows that drastic shortening of the ICD in GlyRs greatly increases agonist efficacy. To our knowledge, this effect has not been reported before, and our data show that it is a robust finding that is not confined to or specific for a single agonist. The enhancement is most conspicuous for partial agonists, is seen in two GlyRs from different species, human and zebrafish, and is consistently detected by two different methods for estimating efficacy, *i.e.* the amplitude of maximum whole cell currents relative to the full agonist glycine and the maximum single channel *P*_open_.

### The gating enhancement is more detectable with partial agonists

We tested a panel of four agonists, glycine, *β*-alanine, taurine, and GABA, whose efficacy values range from very high, for the full agonist glycine, to very low, for the weak partial agonist GABA. ICD shortening in the human GlyR had little or no effect on glycine (+2%), whose maximum *P*_open_ was 0.97 or more in all GlyR tested, but caused pronounced changes for taurine and GABA (+96 and+678%, from 0.46 to 0.90 and 0.09 to 0.70; human GlyR numbers). Reinstating the native ICD in the zebrafish GlyR had little or no effect on glycine responses (–1%), but reduced the maximum *P*_open_ for taurine (–31%) and GABA (–44%, respectively), from 0.95 to 0.66 and from 0.90 to 0.40. This difference in the size of the *P*_open_ effects with agonists of different efficacy is precisely what is expected if our ICD manipulations simply alter the ability of all agonists to gate the receptor, by enhancing channel opening.

In current models of pLGIC activation (17), agonist-bound channels enter an intermediate state (“flip,” closed, but with higher agonist affinity) before opening. Thus, the maximum single channel *P*_open_ depends both on *E*, the opening equilibrium constant and *F*, the “flipping” equilibrium constant.

(Eq. 1) $$\text{Maximum}\;P_{\text{open}}\;=\;\frac{EF}{EF\;+\;F\;+\;1}$$

Agonists with different efficacy differ mostly in their ability to evoke the transition to the intermediate state described by the equilibrium constant *F* (the opening equilibrium constant *E* is similar for different agonists) (18).

Equation 1 can be simplified to,

(Eq. 2) $$\text{Maximum}\;P_{\text{open}}\;=\;\frac{Eff}{Eff\;+\;1}$$

where *Eff* is the overall gating constant for each agonist, *Eff* = (*EF*)/*F*+1).

Equation 2 is the same as the relationship predicted by the simple delCastillo-Katz mechanism (where no activation intermediate is present). Maximum *P*_open_ expressed as a function of log *Eff* gives a sigmoidal curve, and agonists with a maximum *P*_open_ of 0.5 are in the steepest part of the curve, and therefore the most sensitive to changes in *E.*

Global mechanism fits to single-channel data from rat *α*1 GlyR (99% amino acid sequence identity with human *α*1 GlyR) gave estimates for glycine of 8 and 38 for *F* and *E*, respectively, yielding an *Eff* of 34, and a maximum open probability of 0.97 (17, 19). Increasing *Eff* by 10-fold (for instance by increasing *E* by 10-fold) barely shifts this value (by 3% to 0.997).

We do not have *E* and *F* estimates for the other agonists, but we can estimate their *Eff* from the human GlyR single-channel data here, obtaining *Eff* values of 3.5, 0.85, and 0.1 for *β*-alanine, taurine, and GABA, respectively, from the maximum *P*_open_ they produce (0.78, 0.46, and 0.09). If removing the ICD simply increases the opening equilibrium constant *E* for all agonists by 10-fold, it will increase the maximum *P*_open_ for *β*-alanine, taurine, and GABA to 0.97, 0.89, and 0.50, values that are roughly in line with those measured in human GlyR ΔICD (0.92, 0.90, and 0.70). In conclusion, shortening the ICD to a tripeptide linker produced an enhancement in GlyR gating that is likely to be general to all agonists, and is most detectable in those agonists that are not fully efficacious.

We can rank the different channel constructs according to how easy it is for agonists to open them, going from the easiest to open to the most difficult, GlyR_EM_ ≃ zebrafish *α*1 ΔICD > human *α*1 ΔICD + zebrafish TM4 ≃ human *α*1 ΔICD > WT zebrafish *α*1 > WT human *α*1. For the same arguments discussed above, increases in efficacy will be most detectable in the channels that are the most difficult to open.

### The established roles of the GlyR ICD: localization and conductance

Our work confirms the consensus that GlyRs and other pLGICs remain functional and respond to agonists after drastic shortening of the ICD. However, whereas the basic features of channel activation are robust to the loss of the ICD, this domain is known to have multiple effects on pLGIC function. Thus, the ICD provides sites for post-translational modifications (such as ubiquitination and phosphorylation) and directs receptor assembly and trafficking (20, 21, 22, 23, 24, 25, 26). The ICD isolated from the 5-HT_3_ receptor can assemble into stable pentamers (27). For GlyRs, a binding motif in the *β* subunit ICD mediates the receptor interaction with the cytoskeleton via the scaffolding protein gephyrin and ensures the postsynaptic localization and clustering of heteromeric GlyRs (20, 28).

The best recognized direct role of the ICD on channel function is its effect on ion permeation and conductance. This was discovered in the 5-HT_3_ receptor, a cationic pLGIC, where the 20-fold conductance difference between isoforms is due to the presence in the A isoform of three positively charged Arg residues in the amphipathic segment of the ICD that is just before TM4 (29). It was recognized that these residues were likely to line the cytoplasmic portals imaged in the early *Torpedo* nicotinic receptor structures (30, 31) and that they could affect conductance and rectification because they are exposed to the permeation pathway. This effect is general to all pLGICs, and all pLGICs have ICDs that are long enough to form the portals (32), but the effect on conductance is most prominent in cationic channels (33). GlyRs are permeant to anions, and have 8 positively charged residues between the end of the ICD and the early TM4. Mutating up to 7 of these residues to glutamate reduces homomeric GlyR conductance only by one-third and does not affect the EC_50_ of glycine (34). Our constructs preserve 5 of the 8 positive charges (see alignment in Fig. S1).

We have previously measured the slope conductance of rat GlyR bearing the heptapeptide ICD from GLIC and found it unchanged (9), a finding confirmed more recently in GlyR–GLIC chimeras with and without the GlyR ICD (35). This is to be contrasted with data from 5-HT_3_A receptors, where ICD shortening increased conductance by more than 50-fold (7).

### The roles of the ICD: modulation of gating

Our data show that shortening the ICD affects the maximum open probability of GlyRs across a panel of partial agonists. This general increase in efficacy has not been reported before, but other manipulations of the ICD can affect GlyR gating. In particular, phosphorylation of the ICD can change desensitization kinetics, agonist potency, and internalization of pLGICs (36, 37, 38, 39). For GlyR, there is a substantial body of work on the effects of phosphorylation, but the picture that emerges is complex and contradictory. Depending on the type of kinase and the neuronal type involved, phosphorylation can enhance or reduce the effects of submaximal concentrations of glycine. However, there are no data on the effects of phosphorylation on agonist efficacy, *i.e.* maximum responses to partial agonists.

What type of phosphorylation can we expect in our constructs? The WT *α*1 subunits expressed for our recordings are the short isoforms. By analogy to the rat GlyR (40), this human subunit should contain only a PKC site at Ser-391. PKC phosphorylation of this site in mammalian GlyR has been reported to depress (36, 37) or enhance (41, 42, 43) glycine responses. However, Ser residues are not conserved in the zebrafish vs. human ICD (see Fig. S1), so it is difficult to see how the gating enhancement that we observed after shortening the ICD in both the human and zebrafish receptors could be due simply to the removal of phosphorylation sites.

Relatively little is known of how shortening the ICD affects subtler aspects of *α*1 GlyR function. The presence of a native ICD has been reported to be important to the action of positive allosteric modulators on *α*1 GlyRs (44, 45) or GLIC-*α*1 GlyR chimera (Lily) (35), but the mechanism of this effect is not known. Papke and Grossman (46) showed that mutating the ICD of the human *α*1 GlyR (short isoform) or replacing it with the heptapeptide GLIC linker SQPARAA had pronounced effects on the kinetics of desensitization. In 5-HT_3_A receptors, Baptista-Hon *et al.* (32) have shown that one of the several ICD partial truncation constructs tested introduced a phosphorylation site and that phosphorylation of this new site markedly slowed desensitization. In our work we failed to detect marked changes in desensitization, but our whole-cell experiments were not designed to address desensitization kinetics. Experiments with faster concentration jump techniques are needed to measure changes in desensitization robustly. However, it is very unlikely that changes in desensitization can explain the general increase in efficacy we saw after ablating the ICD, because this effect is clearly visible as an increase in single channel maximum *P*_open_, a measurement that factors out desensitization, by excluding desensitized times from the analysis (see Fig. 2*A*).

If a short ICD increases the channel opening equilibrium constant, it must destabilize the resting conformation of the transmembrane gate or stabilize the open conformation of the transmembrane domain. How can that occur? The first possibility is that a drastically shorter ICD introduces an artifactual tension between TM3 and TM4 and that this results in a change in the position of TM3 and its interactions with TM2, the helix that lines the pore and contains the gate. The other possibility is that the native ICD exerts a modulatory effect on pLGIC gating, and by taking different conformations in the open and closed states reduces gating efficacy. In this hypothesis, receptors that lack the native ICD would lack also this modulatory effect on gating. Formulating a precise hypothesis is difficult, because of the lack of structural information on the ICD (see below).

ICD changes can have long range effects on the channel molecule: for instance, PKA phosphorylation of *α*3 GlyR affects the fluorescence signal reported by fluorophores at the top of TM2 and at the tip of the C-loop of the binding site in the extracellular domain (47). In addition to that, the native ICD may also interact with the lipid bilayer, as it contains hot spots for cholesterol binding, but we do not know whether these interactions change with gating (48).

Most of the few structures that have been solved in channel constructs that still contain an ICD are from cationic pLGICs, such as the nicotinic ACh receptor from *Torpedo* (49) and the 5-HT_3_ receptor (50, 51, 52, 53). The information from this work is confined to the sections of the ICD that abut the TM3 at one end and continue into the TM4 at the other. The 5-HT_3_ data support the hypothesis that the ICD takes different conformations in the open and closed states of the channel, as the intracellular ion portals are occluded in the closed configuration by the post-TM3 segment and open as the channel opens (50, 51). It is hard to know whether these findings can be extrapolated to anionic pLGICs, which may lack the amphipathic *α* helix that precedes TM4 (according to secondary structure algorithm predictions (54)). In recent structures of full-length anionic pLGICs such as GABA_A_ receptors (55, 56) and GlyRs (57), densities are still too weak to allow reliable modeling of the ICD.

**Figure 6:**
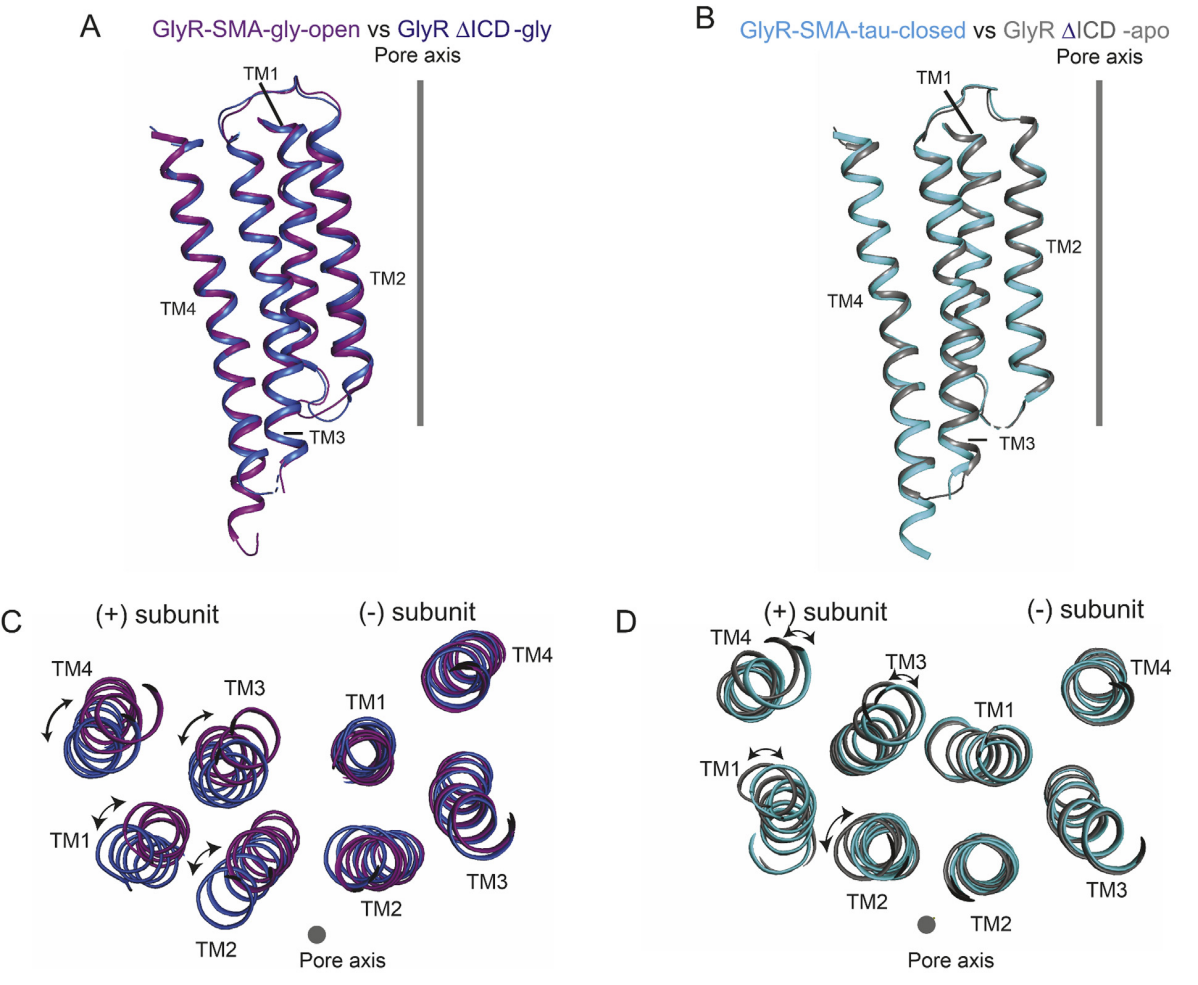
**Conformational changes in the transmembrane domains of the zebrafish full-length GlyR andGlyR** Δ**ICD.** The full-length GlyR and the GlyR ΔICD are in the open (*A* and *C*) and closed (*B* and *D*) states, respectively. *A* and *B,* superposition of transmembrane domains from a single subunit from the full-length GlyR and the GlyR ΔICD from lateral view. *C* and *D,* superimposition of the (–)subunits illustrates the relative movements in the transmembrane domain of the (+)subunit. The view is from the intracellular side. GlyR full-length structures from Yu *et al.* (57), glycine bound open, PDB ID code 6PM6; taurine bound closed, PDB ID code 6PM3. GlyR ΔICD structures from Du *et al.* (2), glycine bound open, PDB ID code 3JAE; closed, PDB ID code 3JAD.

However, the recent GlyR structural data of Yu *et al.* (57) allow us to test the first hypothesis, namely whether shortening the ICD introduces tension between TM3 and TM4 and causes them to reorient their relative position. Fig. 6 shows a comparison between the transmembrane domain of full-length GlyR (*purple* and *cyan*) with that of GlyR ΔICD (ICD replaced with an AGT linker, *blue* and *gray*), in both the closed and open states. Some differences are visible, particularly in the open state structures (*cf*. relative position of the adjacent subunits and the TM2 position), but the positions of the TM3 and TM4 helices within a subunit are likely not affected by the introduction of the shorter ICD linker.

Our work shows that the ICD plays an important role in another key aspect of the *α*1 GlyR function, its maximum response to agonists. When the large native ICD was replaced with a short tripeptide linker, the efficacy of agonists on the *α*1 GlyR dramatically increased. Recent structural information from GlyR suggests that enhanced gating in the receptors with a shortened ICD is likely to stem from the removal of the modulatory action of the native ICD, rather than from an artifact of protein engineering. This opens the possibility that this action of the native ICD can be regulated in neurons by physiological and pathological factors, including phosphorylation and links to the cytoskeleton via gephyrin. Future work will need to explore these possibilities and test the effect of ICD manipulations on the amplitude and time course of glycine-mediated synaptic currents.

## Experimental procedures

### Glycine receptor constructs and expression in HEK 293 cells

The human *α*1 GlyR (accession number P23415-2, *e.g.* short), zebrafish *α*1 GlyR_EM_, and zebrafish *α*1 GlyR were subcloned in the pcDNA3 vector.

We generated a new construct, human *α*1 GlyR ΔICD, by replacing 68 amino acids between Arg-337 and Lys-406 from human *α*1 GlyR (Uniprot accession number P23415-2) with a AGT tripeptide, the same linker used to replace the ICD in the zebrafish *α*1 GlyR_EM_ (Fig. S1). The construct was made by PCR overlap extension. Similarly, the zebrafish *α*1 GlyR ΔICD was constructed by replacing 67 amino acids between Arg-333 and Lys-401 from zebrafish *α*1 GlyR (Uniprot accession number O93430) with AGT tripeptide. The final construct, human GlyR *α*1 + zf TM4, was generated by replacing the ICD from human *α*1 GlyR with AGT tripeptide after which we added the TM4 and C terminus (from Lys-401–Gln-444) from the zebrafish *α*1 WT GlyR. The predicted protein sequence for all constructs used is shown in Fig. S1. The sequence of the reading frame in all constructs was confirmed by Sanger sequencing of the full open frame by Source BioScience LifeSciences (Nottingham, UK).

### Cell culture and transfection

HEK 293 cells (from American Type Culture Collection) were grown at 37 °C in a humidified 95% air, 5% CO_2_ incubator in DMEM (Gibco, 41966029) supplemented with 10% (v/v) heat-inactivated fetal bovine serum, 100 units/ml of penicillin G, 100 *μ*g/ml of streptomycin sulfate (all from Invitrogen). Cells were passaged after reaching 70–80% confluence every 2–3 days, up to 25 times.

For expression, cells were plated on poly-l-lysine–coated glass coverslips (Sigma-Aldrich and VWR, respectively) in 35-mm culture dishes (Scientific Laboratory Supplies) containing 2 ml of DMEM, and then transfected via the calcium phosphate-precipitation method (58) with pcDNA3 plasmids coding for the above mentioned GlyRs.

A plasmid coding for the enhanced green fluorescent protein was added to allow detection of transfected cells. The final DNA mixture contained 2% GlyR cDNA, 20% enhanced green fluorescent protein cDNA, and 78% empty pcDNA3 plasmid. The total amount of the final DNA mixture was 3 *μ*g/plate. The transfection medium was washed off and replaced by fresh medium 4–8 h after transfection. Electrophysiological experiments were performed 1–2 days after transfection.

### Electrophysiology

*Whole-cell recording—*Patch clamp pipettes were pulled from thick-walled borosilicate capillaries (with filament; Harvard Apparatus, Edenbridge, UK) with a Sutter P-97 pipette puller (Sutter Instruments Co.). Pipette tips were fire-polished to obtain a final pipette resistance of 3–5 MΩ. Currents were recorded with an Axopatch 200B amplifier (Molecular Devices). Recordings were pre-filtered at 5 kHz with a 4-pole low-pass Bessel filter (built in the amplifier), digitized at a sampling rate of 20 kHz with a Digidata 1440A (Molecular Devices) and stored on the computer hard drive via the Clampex 10.5 software (Molecular Devices). The bath solution contained (in mm): 20 Na gluconate, 112.7 NaCl, 2 KCl, 2 CaCl_2_, 1.2 MgCl_2_, 10 HEPES, 10 tetraethylammonium chloride, and 30 glucose; the pH was adjusted to 7.4 with NaOH.

The pipettes for whole-cell recording were filled with an internal solution containing (in mm): 101.1 K gluconate, 11 EGTA, 1 CaCl_2_, 1 MgCl_2_, 10 HEPES, 20 TEA-Cl, 2 MgATP, 40 sucrose, and 6 KCl; the pH was adjusted to 7.2 with NaOH. The whole cell macroscopic currents were evoked by U-tube application (59) at the holding potential of −40 mV except in experiments to record GABA responses, where the holding potential was −60 mV to increase the size of the responses. The duration of agonist application was controlled manually and sustained until the response peaked (usually in less than half a second). The position of the U-tube was optimized by applying a diluted bath solution (*e.g.* 50:50, distilled water:bath solution) to the open tip of the recording pipette and measuring the 20–80% rise time of the signal generated by the diluted bath solution. The position of the U-tube was considered acceptable if the response time was less than 20 ms (2–20 ms range). Access resistance for the whole-cell recordings was never higher than 7 mΩ and was compensated by at least 60% and up to 80%.

To monitor run-down/run-up of agonist response, the saturating concentration of agonist was applied every third or fourth application. The recording was accepted for analysis if the run-down/run-up was less than 30%. To normalize responses to *β*-alanine, taurine and GABA to the glycine maximum current (*I*_ago_/*I*_gly_), a saturating concentration of glycine (10 mm) was applied at the beginning and end of the experiment. For the analysis, whole cell recordings were filtered at 1 kHz, and the peak of response was determined in Clampfit 10.5 software (Molecular Devices). The data were analyzed by custom made analysis software (CVFIT version 1.0.0-alpha; https://github.com/DCPROGS/CVFIT/releases/tag/v1.0.0-alpha)^1^ and fitted with the Hill equation,

(Eq. 3) $$y\;=\;y_{\max}\frac{{\left[\text{A}\right]}^{n_{\text{H}}}}{{\left[\text{A}\right]}^{n_{\text{H}}}\;+\;EC_{50}^{n_{\text{H}}}}$$

where *y*_max_ is the maximum response current, *n*_H_ is the Hill coefficient, and EC_50_ is the agonist concentration required to evoke 50% of the maximum response.

A full dose-response curve was obtained in each cell. The responses were normalized to the fitted maximum in each cell and subsequently pooled and refitted with the Hill equation for display.

*Single-channel recording—*Pipette tips were coated with Sylgard (Dow Corning) and heat polished to a final resistance of 8–12 mΩ. Pipettes were filled with extracellular solution (the same as the bath solution used for whole cell recordings) to which agonists were added from stock solutions to the desired concentration. Cell-attached recordings were obtained with an Axopatch 200B (Molecular Devices) amplifier at the +100 mV holding voltage, prefiltered at 10 kHz with the built-in 4-pole Bessel filter, and digitized at 100 kHz with a Digidata 1440A (Molecular Devices). For the purpose of analysis, recordings were resampled at 33.3 kHz and filtered at 3 kHz Gaussian filter by using Clampex 10.5 software.

Clusters of GlyR activity were accepted for analysis only if they were longer than 100 ms and separated by at least 100 ms of shut time. Openings were idealized by threshold crossing in Clampfit 10.5 and the *P*_open_ was calculated as the ratio between the time during which the channel was open and the total length of the cluster. In the cluster *P*_open_ plots (OriginPro 2019; OriginLab), the box shows the 25th and 75th percentiles, and the whiskers extend to the furthest point that falls within 1.5 times of the interquartile range from the 25th to 75th percentile.

In channels with shortened ICDs, we observed transitions to a subconductance level with an amplitude of ∼1 pA (*cf*. 4–6 pA for the full openings; Fig. S2 shows an example for the human GlyR ΔICD). Dwells in these subconductance levels were treated as closures because of the threshold-crossing analysis. These events are not very frequent; treating them as open would increase the open probability measured in GlyR with engineered ICD by 5–6% and would not change the gist of our results.

All solutions were prepared from bi-distilled water to reduce contaminant glycine and filtered through a 0.2-*μ*m Cyclopore track-etched membrane (GE Healthcare) to remove impurities. Agonists were purchased from Fluka-Sigma and tested for glycine contamination by HPLC assay at a concentration of 300 mm. The GABA and taurine samples were found to contain contaminant glycine at 0.5 and 0.4 *μ*m, respectively. These concentrations of glycine are well below those able to evoke a response in the GlyRs used in this work (the lowest concentration being 30 *μ*m for zebrafish *α*1 GlyR_EM_) and therefore agonists were not further purified for whole cell recordings. Nevertheless, for the single-channel recordings, we used purified GABA and taurine, obtained by re-crystallizing three times from aqueous ethanol. The purified agonist solutions were tested again for glycine contamination by HPLC, which confirmed that the glycine contamination had been eliminated.

### Statistical testing

Results are reported as the mean ± S.D., where *n* represents number of cells, clusters, or patches as indicated. A nonparametric randomization test (two-tail, unpaired; 10,000 iterations) (DCStats version 0.3.1-alpha; https://github.com/DCPROGS/DCSTATS/releases/tag/v.0.3.1-alpha)^1^ and we set the threshold at a more stringent level *p* < 0.005 to allow for multiple comparisons (this would correspond to a Bonferroni correction for 10 comparisons and a *p* < 0.05).

## Data availability

The atomic coordinates and structure factors (codes 6PM3 and 6PM6) have been deposited in the Protein Data Bank (http://wwpdb.org/).

## Supporting information

This article contains supporting information.

## Conflict of interest

The authors declare that they have no conflicts of interest with the contents of this article.

## Footnotes

1 Please note that the JBC is not responsible for the long-term archiving and maintenance of this site or any other third party hosted site.
