# Supplementary material for: The intracellular domain of homomeric glycine receptors modulates agonist efficacy
Source: J Biol Chem. 2021 Feb 20;296:100387. doi: 10.1074/jbc.RA119.012358 (PMC7995613; doi:10.1074/jbc.RA119.012358)
Supplement: Figures and Tables [file mmc1.pdf]

The intracellular domain of homomeric glycine receptors modulates agonist efficacy

Josip Ivica[1], Remigijus Lape[1], Vid Jazbec[2], Jie Yu[3], Hongtao Zhu[3], Eric Gouaux[4], Matthew G. Gold[1] and Lucia G. Sivilotti[1]*

From the [1]Department of Neuroscience, Physiology and Pharmacology, Division of Biosciences, University College London, Gower St, London WC1E 6BT, United Kingdom; [2]Department of Synthetic Biology and Immunology, National Institute of Chemistry, Hajdrihova 19, 1000 Ljubljana, Slovenia. [3]Vollum Institute, Oregon Health & Science University, Portland, Oregon 97239, USA, [4]Howard Hughes Medical Institute, Oregon Health & Science University, Portland, Oregon 97239, USA.

Supporting Information

Figure S1

Figure S2

Table S1

```
                                     Signal peptide
human α1 GlyR             MYSFNTLRLYLWETIVFFSLAASKEAEAARSAPKPMSPSDFLDKLMGRTSGYDARIRPNF 60
zebrafish α1 GlyR         ---MFALGIYLWETIVFFSLAASQQAA-ARKAASPMPPSEFLDKLMGKVSGYDARIRPNF 56
zebrafish α1 GlyREM       ---MFALGIYLWETIVFFSLAASQQAA-A--------PSEFLDKLMGKVSGYDARIRPNF 48
zebrafish α1 GlyR Δ ICD   ---MFALGIYLWETIVFFSLAASQQAA-ARKAASPMPPSEFLDKLMGKVSGYDARIRPNF 56
human α1 GlyR Δ ICD       MYSFNTLRLYLWETIVFFSLAASKEAEAARSAPKPMSPSDFLDKLMGRTSGYDARIRPNF 60
hu α1 GlyR ΔICD + zf TM4  MYSFNTLRLYLWETIVFFSLAASKEAEAARSAPKPMSPSDFLDKLMGRTSGYDARIRPNF 60
                                                        # ##  #  #       ##

human α1 GlyR             KGPPVNVSCNIFINSFGSIAETTMDYRVNIFLRQQWNDPRLAYNEYPDDSLDLDPSMLDS 120
zebrafish α1 GlyR         KGPPVNVTCNIFINSFGSIAETTMDYRVNIFLRQQWNDPRLAYSEYPDDSLDLDPSMLDS 116
zebrafish α1 GlyREM       KGPPVNVTCNIFINSFGSIAETTMDYRVNIFLRQQWNDPRLAYSEYPDDSLDLDPSMLDS 108
zebrafish α1 GlyR Δ ICD   KGPPVNVTCNIFINSFGSIAETTMDYRVNIFLRQQWNDPRLAYSEYPDDSLDLDPSMLDS 116
human α1 GlyR Δ ICD       KGPPVNVSCNIFINSFGSIAETTMDYRVNIFLRQQWNDPRLAYNEYPDDSLDLDPSMLDS 120
hu α1 GlyR ΔICD + zf TM4  KGPPVNVSCNIFINSFGSIAETTMDYRVNIFLRQQWNDPRLAYNEYPDDSLDLDPSMLDS 120
                                 #                                   #

human α1 GlyR             IWKPDLFFANEKGAHFHEITTDNKLLRISRNGNVLYSIRITLTLACPMDLKNFPMDVQTC 180
zebrafish α1 GlyR         IWKPDLFFANEKGANFHEVTTDNKLLRISKNGNVLYSIRITLVLACPMDLKNFPMDVQTC 176
zebrafish α1 GlyREM       IWKPDLFFANEKGANFHEVTTDNKLLRISKNGNVLYSIRITLVLACPMDLKNFPMDVQTC 168
zebrafish α1 GlyR Δ ICD   IWKPDLFFANEKGANFHEVTTDNKLLRISKNGNVLYSIRITLVLACPMDLKNFPMDVQTC 176
human α1 GlyR Δ ICD       IWKPDLFFANEKGAHFHEITTDNKLLRISRNGNVLYSIRITLTLACPMDLKNFPMDVQTC 180
hu α1 GlyR ΔICD + zf TM4  IWKPDLFFANEKGAHFHEITTDNKLLRISRNGNVLYSIRITLTLACPMDLKNFPMDVQTC 180
                                        #   #          #            #

human α1 GlyR             IMQLESFGYTMNDLIFEWQEQGAVQVADGLTLPQFILKEEKDLRYCTKHYNTGKFTCIEA 240
zebrafish α1 GlyR         IMQLESFGYTMNDLIFEWDEKGAVQVADGLTLPQFILKEEKDLRYCTKHYNTGKFTCIEA 236
zebrafish α1 GlyREM       IMQLESFGYTMNDLIFEWDEKGAVQVADGLTLPQFILKEEKDLRYCTKHYNTGKFTCIEA 228
zebrafish α1 GlyR Δ ICD   IMQLESFGYTMNDLIFEWDEKGAVQVADGLTLPQFILKEEKDLRYCTKHYNTGKFTCIEA 236
human α1 GlyR Δ ICD       IMQLESFGYTMNDLIFEWQEQGAVQVADGLTLPQFILKEEKDLRYCTKHYNTGKFTCIEA 240
hu α1 GlyR ΔICD + zf TM4  IMQLESFGYTMNDLIFEWQEQGAVQVADGLTLPQFILKEEKDLRYCTKHYNTGKFTCIEA 240
                                            # #
                                     TM1                              TM2
human α1 GlyR             RFHLERQMGYYLIQMYIPSLLIVILSWISFWINMDAAPARVGLGITTVLTMTTQSSGSRA 300
zebrafish α1 GlyR         RFHLERQMGYYLIQMYIPSLLIVILSWVSFWINMDAAPARVGLGITTVLTMTTQSSGSRA 296
zebrafish α1 GlyREM       RFHLERQMGYYLIQMYIPSLLIVILSWVSFWINMDAAPARVGLGITTVLTMTTQSSGSRA 288
zebrafish α1 GlyR Δ ICD   RFHLERQMGYYLIQMYIPSLLIVILSWVSFWINMDAAPARVGLGITTVLTMTTQSSGSRA 296
human α1 GlyR Δ ICD       RFHLERQMGYYLIQMYIPSLLIVILSWISFWINMDAAPARVGLGITTVLTMTTQSSGSRA 300
hu α1 GlyR ΔICD + zf TM4  RFHLERQMGYYLIQMYIPSLLIVILSWISFWINMDAAPARVGLGITTVLTMTTQSSGSRA 300
                                                     #
                                              TM3                  ICD
human α1 GlyR             SLPKVSYVKAIDIWMAVCLLFVFSALLEYAAVNFVSRQHKELLRFRRKRRHHKEDEAGEG 360
zebrafish α1 GlyR         SLPKVSYVKAIDIWMAVCLLFVFSALLEYAAVNFIARQHKELLRFQRRRRHLKEDEAGDG 356
zebrafish α1 GlyREM       SLPKVSYVKAIDIWMAVCLLFVFSALLEYAAVNFIAR---------AGT----------- 327
zebrafish α1 GlyR Δ ICD   SLPKVSYVKAIDIWMAVCLLFVFSALLEYAAVNFIAR---------AGT----------- 335
human α1 GlyR Δ ICD       SLPKVSYVKAIDIWMAVCLLFVFSALLEYAAVNFVSR---------AGT----------- 339
hu α1 GlyR ΔICD + zf TM4  SLPKVSYVKAIDIWMAVCLLFVFSALLEYAAVNFVSR---------AGT----------- 339
                                                            ##
                                                                        TM4
human α1 GlyR             RFNFSAYGMGPACLQAKDGISVKGANNSNTTNPPPAPSKSPEEMRKLFIQRAKKIDKISR 420
zebrafish α1 GlyR         RFSFAAYGMGPACLQAKDGMAIKGNNNNAPTST-NPPEKTVEEMRKLFISRAKRIDTVSR 415
zebrafish α1 GlyREM       ---------------------------------------------KLFISRAKRIDTVSR 343
zebrafish α1 GlyR Δ ICD   ---------------------------------------------KLFISRAKRIDTVSR 351
human α1 GlyR Δ ICD       ---------------------------------------------KLFIQRAKKIDKISR 355
hu α1 GlyR ΔICD + zf TM4  ---------------------------------------------KLFISRAKRIDTVSR 355
                                                                           #   #  ##
                                              C-term
human α1 GlyR             IGFPMAFLIFNMFYWIIYKIVRREDVHNQ 449
zebrafish α1 GlyR         VAFPLVFLIFNIFYWITYKIIRSEDIHKQ 444
zebrafish α1 GlyREM       VAFPLVFLIFNIFYWITYKLVPRGS---- 368
zebrafish α1 GlyR Δ ICD   VAFPLVFLIFNIFYWITYKIIRSEDIHKQ 380
human α1 GlyR Δ ICD       IGFPMAFLIFNMFYWIIYKIVRREDVHNQ 384
hu α1 GlyR ΔICD + zf TM4  VAFPLVFLIFNIFYWITYKIIRSEDIHKQ 384
                          ##  ##     #    #   # #  # #
```

Figure S 1. Clustal W alignment of GlyR constructs used in this work. Differences between human α1 GlyR and zebrafish α1 GlyR outside the ICD are marked with #. The amino acids involved in glycine binding are highlighted in green. The AGT linker used to replace the native ICD in some of the GlyR constructs is the same as used by Du.et al. for zebrafish α1 GlyR$_{EM}$ and is highlighted in yellow (1).

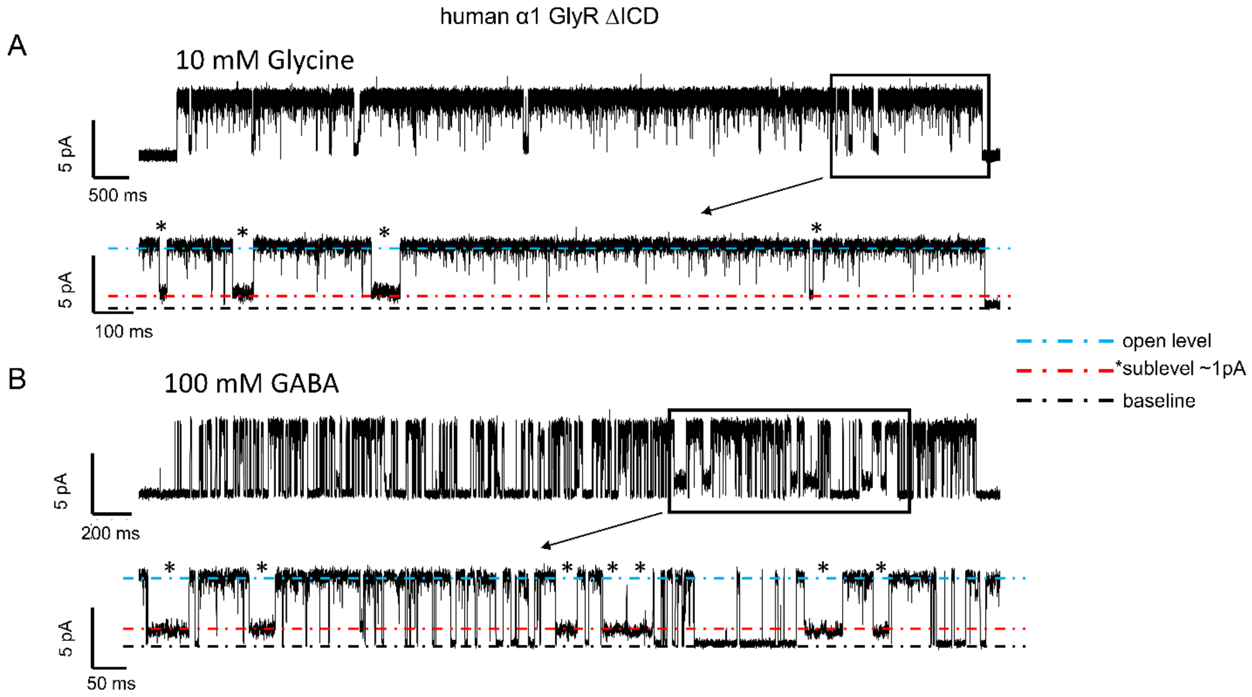

Figure S 2. ***Sublevel of amplitude observed in human a1 GlyR Δ ICD.*** A Cluster of single channel activity evoked by saturating concentration of Glycine (top trace). The area enclosed with a rectangle is presented in the bottom trace. The events that are not reaching full closure are annotated with *. B) Cluster of single channel activity evoked by saturating concentration of GABA (top trace). The area enclosed with a rectangle is presented in the bottom trace. The events that are not reaching full closure are annotated with *.

Table S 1. Pairwise comparison of parameters from whole-cell and single-channel recording. P values are estimated using two tail randomization test (10000 randomization iterations).

GlyR pair	Agonist (p value)				
	parameter	GABA	Taurine	Balanine	Glycine
**human α1 GlyR & zebrafish α1 GlyR$_{EM}$**	EC_{50}	$<10^{-4}$	0.0004	0.0006	0.0010
	I_{rel}	$<10^{-4}$	$<10^{-4}$	$<10^{-4}$	/
	$maxP_{oepn}$	$<10^{-4}$	$<10^{-4}$	$<10^{-4}$	0.3396
**human α1 GlyR & human α1 GlyR Δ ICD**	EC_{50}	0.0093	0.0146	0.0235	0.0049
	I_{rel}	0.0001	$<10^{-4}$	0.0061	/
	$maxP_{oepn}$	$<10^{-4}$	$<10^{-4}$	0.0004	0.3397
**zebrafish α1 GlyR & zebrafish α1 GlyR$_{EM}$**	EC_{50}	$<10^{-4}$	0.0037	0.0344	0.0111
	I_{rel}	$<10^{-4}$	0.0008	0.0003	/
	$maxP_{open}$	$<10^{-4}$	$<10^{-4}$	0.0503	0.2548
**human α1 GlyR & zebrafish α1 GlyR**	EC_{50}	0.0006	0.0004	0.0003	0.1384
	I_{rel}	$<10^{-4}$	0.0266	0.0143	/
	$maxP_{oepn}$	$<10^{-4}$	$<10^{-4}$	0.0006	0.6696
**human α1 GlyR Δ ICD& zebrafish α1 GlyR$_{EM}$**	EC_{50}	$<10^{-4}$	0.0002	0.0014	0.0084
	I_{rel}	$<10^{-4}$	0.0036	0.0004	/
	$maxP_{oepn}$	$<10^{-4}$	0.0203	0.0151	0.1084
**human α1 GlyR Δ ICD & huα1 GlyR Δ ICD+ TM4 zf**	EC_{50}	0.0009	0.9264	0.1040	0.0223
	I_{rel}	0.2456	0.7757	0.9124	/
	$maxP_{open}$	0.6194	0.7895	0.2263	0.0054
**zebrafish α1 GlyR$_{EM}$ & zebrafish α1 GlyR Δ ICD**	$maxP_{open}$	0.8052	0.5591	0.6066	0.0454

References

1. Du, J., Lu, W., Wu, S., Cheng, Y., and Gouaux, E. (2015) Glycine receptor mechanism elucidated by electron cryo-microscopy. *Nature* **526**, 224-229
